# Complex Origins and History of the Relict Fennoscandian Ringed Seals

**DOI:** 10.1002/ece3.71067

**Published:** 2025-03-04

**Authors:** Morten Tange Olsen, Ari Löytynoja, Mia Valtonen, Steen W. Knudsen, Sofie Bang, Casper Gunnersen, Aqqalu Rosing‐Asvid, Steven H. Ferguson, Rune Dietz, Kit M. Kovacs, Christian Lydersen, Jukka Jernvall, Petri Auvinen, Anders Galatius

**Affiliations:** ^1^ Section for Molecular Ecology and Evolution, Globe Institute University of Copenhagen Copenhagen K Denmark; ^2^ Section for Marine Mammal Research, Department of Ecoscience Aarhus University Roskilde Denmark; ^3^ Institute of Biotechnology University of Helsinki Helsinki Finland; ^4^ Wildlife Ecology Group Natural Resources Institute Finland Helsinki Finland; ^5^ NIVA Denmark Water Research Copenhagen Denmark; ^6^ Department of Birds and Mammals Greenland Institute of Natural Resources Nuuk Greenland; ^7^ Fisheries and Oceans Canada Winnipeg Manitoba Canada; ^8^ Norwegian Polar Institute Fram Centre Tromsø Norway; ^9^ Department of Geosciences and Geography University of Helsinki Helsinki Finland

**Keywords:** climate change, mitogenome, morphometrics, phylogeography, quaternary

## Abstract

Spatiotemporal environmental heterogeneity is a major evolutionary driver, which can cause profound phylogeographic complexity, particularly at the periphery of species ranges. Ringed seals display a highly disjoint distribution, occurring in high abundance throughout the circumpolar Arctic, as well as in the Baltic Sea, Lake Saimaa and Lake Ladoga. These relict Fennoscandian ringed seals were traditionally regarded as originating from a single colonisation event after the Last Glacial Maximum (LGM), but recent studies have challenged this perception. Here, we analyse 246 mitogenomes and 180 skulls to unravel the diversity and spatiotemporal pattern of diversification in Fennoscandian ringed seals. Contrary to previous assumptions, our results reveal a complex evolutionary history characterised by pre‐LGM diversification from Arctic ringed seals and possibly several Fennoscandian colonisation events. We hypothesise that Saimaa seals originate from Arctic ringed seals, from which they diverged prior to their arrival in Lake Saimaa. Ladoga seals appear to also originate from the Arctic, with secondary colonisation events from paleo‐Skagerrak–Kattegat–Baltic, while the Baltic ringed seals have mixed evolutionary origins. Lake Saimaa and, to some extent, Lake Ladoga ringed seals have experienced a loss of diversity and evolved divergent skull morphologies, likely as a result of colonisation bottlenecks, isolation and dietary specialisation, while Baltic Sea ringed seals have retained remarkably high levels of genetic and morphological diversity. Our study supports the classification of Saimaa, Ladoga and Baltic ringed seals as distinct taxa and highlights the need for management and conservation efforts to mitigate cumulative impacts of human activities and climate change on Fennoscandian ringed seals.

## Graphical abstract

We combine mitogenome and morphometric analyses to formulate novel hypotheses on the evolutionary origin and drivers of the genetic and phenotypic diversity of Fennoscandian ringed seals. Our research highlights the complex origins of the three subspecies, of relevance for a broader understanding of the Quaternary history of northern Europe and in particular its colonisation by flora and fauna.

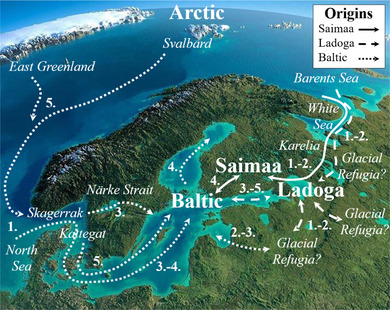

## 1 Introduction

The distribution and genetic diversity of the Earth's flora and fauna were influenced greatly by Late Quaternary environmental fluctuations (Hewitt 2000). Many terrestrial species follow a general pattern, in which cold‐adapted species expand their range towards lower latitudes during glacial periods and retract to high‐latitude (or alpine) refugia during interglacial periods (Stewart et al. 2010). However, spatiotemporal environmental heterogeneity and physical geography introduce the possibility of creating refugia at high latitudes during glacial periods, and at low latitudes during interglacial periods, thus increasing the phylogeographic complexity and overall genotypic and phenotypic diversity of species. In particular, this seems to be the case for many Arctic and boreal marine species, with cryptic diversity and complex phylogeography reported for invertebrates (Tempestini et al. 2020), fish (Madsen et al. 2016; Jacobsen et al. 2022), pinnipeds (Carr et al. 2015; Rosing‐Asvid et al. 2023; Ruiz‐Puerta et al. 2023) and cetaceans (Louis et al. 2020; Skovrind et al. 2021; Olsen et al. 2022). Identifying such diversity and understanding how it was formed is central to our understanding of a species' ecology and evolution, as well as for formulating efficient management and conservation strategies to meet the challenges of the biodiversity and climate crises.

**FIGURE 1:**
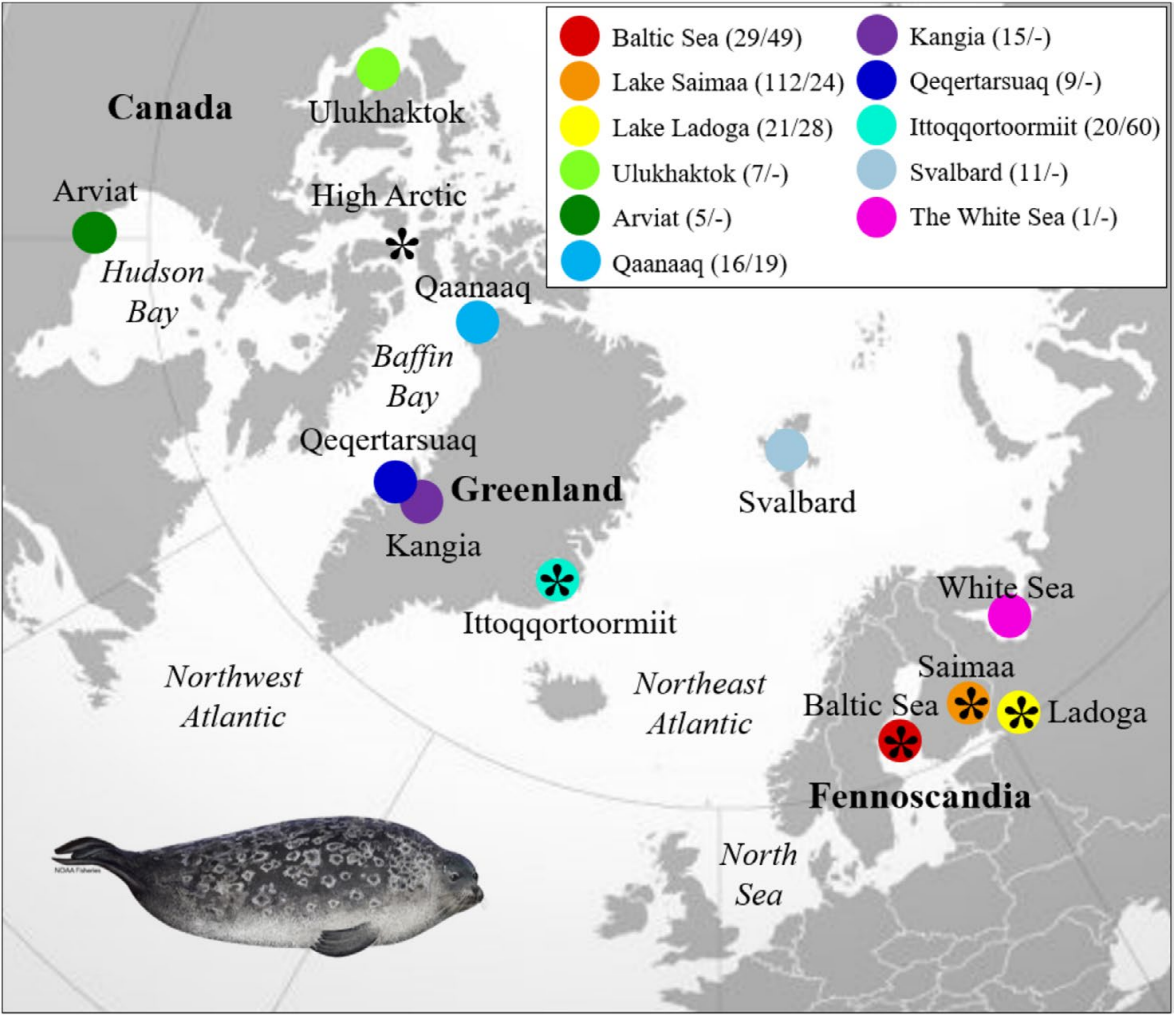
Fennoscandian and Arctic ringed seal sampling localities. The study included 246 mitogenomes from 11 localities (circles) and 180 skulls from five regions (asterisk), representing most of the species' range in Canada, Greenland, Svalbard and northern Europe. Sample sizes for each locality are listed for mitogenomes and skulls, when applicable. The skulls representing the ‘High Arctic’ are listed under Qaanaaq but originate from multiple distinct localities in the region of northwest Greenland and the Canadian High Arctic. Ringed seal illustration courtesy of NOAA Fisheries.

The ringed seal ( *Pusa hispida* , (Schreber 1775)) is one of the most widespread mammals in the northern hemisphere and a key species in the marine food web (Durner et al. 2017; Hamilton et al. 2017). The species is highly adapted to polar conditions, capable of maintaining breathing holes in sea ice and hence occupying areas of seasonal or continuous ice cover, allowing it to overwinter in the High Arctic and possibly persist at high latitudes during glacial periods (Davies 1958; Rosing‐Asvid et al. 2023). The reproductive success of ringed seals is tied to the stability of sea ice and adequate snow cover, with birth and lactation taking place in subnivean (snow) lairs on the sea ice (McLaren 1958; Smith and Lydersen 1991; Hammill 2009). Intriguingly, while the Arctic subspecies ( *Pusa hispida hispida* ) occurs throughout the circumpolar Arctic, contemporary ringed seals have a disjoint distribution with multiple additional ‘relict’ subspecies inhabiting sub‐Arctic and cold‐temperate environments of the Baltic Sea (*P. h. botnica*), Lake Ladoga (*P. h. ladogensis*), and Lake Saimaa (*P. h. saimensis*), as well as the Sea of Okhotsk (*P. h. ochotensis*) (Davies 1958), (Figure 1).

The Fennoscandian ringed seals in the Baltic Sea, Lake Ladoga and Lake Saimaa are generally assumed to have colonised their current ranges after the LGM from glacial refugia in the North Sea–Skagerrak–Kattegat region of northern Europe (Davies 1958; Palo et al. 2001; Sommer and Benecke 2003; Valtonen et al. 2012; Ukkonen et al. 2014). This could have occurred via river outlets through central Sweden (Närke Strait) and/or the Danish Straits (Dana River) into the Baltic Sea and further into Lake Saimaa and Lake Ladoga, with each of the three Fennoscandian taxa becoming isolated as the main species distribution retracted north and isostatic rebound formed the Ladoga and Saimaa lakes. However, genetic studies have suggested that periodic gene flow has occurred from both the Arctic and Lake Ladoga to the Baltic Sea (Palo et al. 2001; Martinez‐Bakker et al. 2013; Nyman et al. 2014), and recent demographic reconstructions indicate that the origin of the Saimaa seal evolutionary lineage may predate the LGM (Löytynoja et al. 2023). Thus, the colonisation of Fennoscandia by ringed seals may be more complex than first assumed, perhaps involving refugia in glacial lakes at the edge of the Fennoscandian Ice Sheet and/or interglacial dispersal routes directly from the Arctic through Karelia (Heino et al. 2023).

In addition to affecting genetic diversity, spatiotemporal environmental and geographical heterogeneity may have influenced the ringed seal's phenotypic diversity. In particular, differences in marine and freshwater habitat size and characteristics, such as bathymetry, temperature and salinity, are expected to affect the available prey base and in turn drive the evolution of different foraging strategies and morphologies. For example, analyses of body size variation among Arctic ringed seals suggest that pack‐ice seals generally are smaller than fast ice ringed seals and these differences appear to also be reflected in their skull morphology (Finley et al. 1983). Ringed seals in eastern Canada show a marked latitudinal gradient in body size, growth rates and life history (Ferguson et al. 2018, 2019, 2020). At a wider geographical scale, the body size of animals in West Greenland and the High Arctic (Northwest Greenland and Northeast Canada), and to some extent Svalbard, appears to be larger than the average, while animals in Alaska and the White Sea are smaller (Kovacs et al. 2021). Likewise, analyses of Fennoscandian ringed seals suggest that Ladoga ringed seals are smaller than other ringed seals, Saimaa ringed seals are similar to medium‐sized Arctic ringed seals, but smaller than those in the Baltic Sea, which are comparable in size to the large ringed seals found in the Canadian High Arctic (Helle 1979; McLaren 1993; Auttila et al. 2016; Ferguson et al. 2018). Similarly, the skull morphology appears to vary among Fennoscandian and Arctic ringed seals, with Saimaa and Ladoga seals, but not Baltic seals, differing from each other and Arctic ringed seals (Amano et al. 2002). Nevertheless, it is unclear if these examples of body size and skull shape variation reflect phenotypic plasticity in response to environmental conditions, genetic adaptations or a combination of the two (but see Rosing‐Asvid et al. 2023).

Thus, although Fennoscandian and Arctic ringed seals have previously been subject to analyses of both genetic data (Palo et al. 2001, 2003; Davis et al. 2008; Valtonen et al. 2012; Martinez‐Bakker et al. 2013; Nyman et al. 2014; Heino et al. 2023; Rosing‐Asvid et al. 2023; Sundell et al. 2023) and morphological data (Helle 1979; Finley et al. 1983; McLaren 1993; Amano et al. 2002; Auttila et al. 2016; Ferguson et al. 2018; Kovacs et al. 2021), several questions about their evolutionary history remain unanswered. Here, we combine analyses of 246 mitogenomes with morphological measurements of 180 skulls to shed new light on the phylogeography of Fennoscandian ringed seals and their genotypic and phenotypic diversity. Specifically, we seek to: (i) use mitogenomic data to generate hypotheses regarding the timing and route(s) of ringed seal colonisation of Fennoscandia and whether this occurred as a single stepwise event or through repeated colonisation waves; (ii) assess putative differences in skull morphology to infer the role of dietary specialisation in the diversification of Fennoscandian ringed seals; and (iii) discuss the implications of our findings for the status and fate of Fennoscandian ringed seals in a warming world. Compared to previous work, our study expands the genetic and morphological sample size from Fennoscandian ringed seals and substantially increases the genetic sample size from the Atlantic Arctic. Notably, we fill a major geographical gap in previous studies by including mitogenome and morphological data from East Greenland ringed seals, which are among the Arctic ringed seals that have the shortest (waterway) distance to Fennoscandia and hence might be a possible source of founders and migrants.

## 2 Materials and Methods

### 2.1 Mitogenome Analyses

#### 2.1.1 Sampling, DNA Extraction and Sequencing

Ringed seal tissue samples for mitogenome analyses were collected from ringed seals in Fennoscandia and across the Atlantic Arctic (Figure 1; Table 1). DNA from Svalbard, the Baltic Sea, Lake Ladoga and Lake Saimaa was extracted using NucleoSpin Tissue Kit (Macherey‐Nagel) and sequenced as described in Savriama et al. (2018), Löytynoja et al. (2023) and Sundell et al. (2023), while DNA was extracted from the Greenlandic and Canadian samples using the Thermo Scientific KingFisher Cell and Tissue DNA Kit (Germany), as described in Rosing‐Asvid et al. (2023). DNA libraries from the Greenlandic and Canadian samples were built using the blunt end single tube (BEST) protocol (Carøe et al. 2018) and sequenced on the Illumina HiSeq4000 platform using paired end 150 bp at the National High‐throughput DNA Sequencing Centre, University of Copenhagen, Denmark.

**TABLE 1:** Genetic summary statistics for Fennoscandian and Arctic ringed seals, as well as each Arctic locality separately.

Area	*N*	*h*	*P*	*H*_d_	*π*	*S*	*K*
Subspecies							
Atlantic Arctic	84	83	0.000	1.000	0.938	972	100.5
Baltic Sea	29	26	0.109	0.995	0.847	286	90.7
Lake Ladoga	21	15	0.074	0.962	0.313	168	33.6
Lake Saimaa	112	7	0.070	0.697	0.044	14	4.7
Atlantic Arctic localities							
Ittoqqortoormiit	20	20	—	1.00	0.969	577	103.8
Qaanaaq	16	16	—	1.00	0.905	441	97.0
Qeqertarsuaq	9	9	—	1.00	1.015	335	108.7
Kangia	15	15	—	1.00	0.949	396	101.6
Ulukhaktok	7	7	—	1.00	1.074	294	115.1
Arviat	5	5	—	1.00	0.894	178	95.8
Svalbard	11	11	—	1.00	0.865	361	92.7
White Sea	1	—	—	—	—	—	—
Total	246	131		0.937	0.762	1054	81.6

*Note:* The mitogenome diversity of Baltic and Arctic ringed seals is extremely high, whereas in particular Saimaa seals are characterised by low levels of mitogenome diversity.

Abbreviations: *h* = haplotypes; *H*_d_ = haplotype diversity; *K* = Average number of differences; *N* = Number of sequences; *P* = probably of *h* given *H* and theta, based on Ewens 1972; *S* = segregating sites; π = nucleotide diversity.

#### 2.1.2 Data Filtering and Mapping

Sequence reads were mapped to a Baltic Sea ringed seal mitogenome (NC_008433; Arnason et al. 2006) using Geneious Prime 2019.0.4 (https://www.geneious.com). We focused our analyses on the 12 coding regions (i.e., ND1, ND2, COX1, COX2, ATP8, ATP6, COX3, ND3, ND4L, ND4, ND5 and CYTB) to obtain a dataset comprising 246 mitogenomes at a length of 10,713 bp (File S1). While this reduction of the data might deflate estimates of mitogenome diversity, it should still allow for inferring the phylogeography of ringed seals.

#### 2.1.3 Haplotype Diversity and Differentiation

DNAsp (Rozas et al. 2017) was used for calculating the number of variable sites (S), haplotypes (h), haplotype diversity (H_d_), nucleotide diversity (π), and average number of differences (K) for each location, as well as for the Atlantic Arctic as a whole. PopArt (Leigh and Bryant 2015) was used to create a median‐joining network (Bandelt et al. 1999) for graphically displaying haplotype richness and diversity. Finally, genetic differentiation between sample sites was estimated by *K*_ST_ (Hudson et al. 1992) using 1000 permutations to determine the significance of the observed values. Moreover, we estimated the net number of nucleotide substitutions per site, *d*_A_ (Nei 1987, equation 10.21) for a tentative evaluation of taxonomic classification (Morin et al. 2023).

#### 2.1.4 Phylogeography

The phylogenetic analyses were conducted on a reduced dataset of 141 ringed seal mitogenomes in which identical haplotypes from the Saimaa seal were removed as they are phylogenetically uninformative, and removing them eases computation and visualisation. We used BEAST2.5.2. (Bouckaert et al. 2019) to construct a phylogenetic tree and estimate divergence times among major ringed seal lineages. First, the data were partitioned into both codon position and individual genes with the time‐tree and clock model linked for all the partitions. As initial trials with a relaxed clock and a random local clock had difficulties stabilising for the posteriors, priors and likelihoods, we used a strict clock model, no calibration of nodes and a HKY nucleotide substitution model with estimated frequencies, a fixed mutation rate and a Gamma category count of 4. The tree prior was set to Birth Death Model with a 1/x distribution, with a chain length at 40,000,000 and logging of every 1000th tree, resulting in 40,000 trees. Tracer (Rambaut et al. 2018) was used to explore the outputs from the Bayesian analysis and to assess that the effective sample size (ESS) was > 200 for all parameters. The tree file was processed in TreeAnnotator v2.5.2, with a 10% burn‐in, a posterior probability limit of 0, using Maximum clade credibility with node heights set to mean heights. The resulting tree was visualised in Figtree v. 1.4.4 (Rambaut 2006–2018). In addition, to supplement the Bayesian phylogeny, we constructed a maximum likelihood tree using PhyML with a GTR model, four substitution rate categories and 1000 bootstrap iterations (Guindon et al. 2010).

In order to rescale the root (‘crown age’) of our ringed seal Bayesian phylogeny, we performed a time‐calibrated multi‐species Bayesian phylogenetic analysis in BEAST2 using an alignment of mitogenomes from eight related phocid seal species with 36 of our ringed seal mitogenomes representing the main evolutionary lineages A–H identified in the ringed seal phylogenetic analysis (File S2). We linked clock and tree models, used an HKY model with four gamma categories, and as priors a Birth–Death model with gamma shaped birth and death rates. To time the tree, we defined prior calibration points for four monophyletic groups: Phocini (mean = 5.215 Mya; 95%HPD = 2.24–8.00 Mya; normal with sigma = 1.7, offset = 0.0), Phocina (mean = 2.375 Mya; 95%HPD = 1.14–3.61 Mya; normal with sigma = 0.75, offset = 0.0), ringed seal and Baikal seal (mean = 1.89 Mya; 95%HPD = 1.28–2.55; normal with sigma = 0.4, offset = 0.0) and finally our 36 ringed seal mitogenomes to obtain a ringed seal crown age (median = 0.882; 95%HPD = 0.265–2.56; gamma with offset = 0.2; alpha = 1.2; beta = 0.7). These calibration point priors are based on the divergence times inferred from pinniped‐specific mitogenome analyses (Fulton and Strobeck 2010), which we preferred over the generally much older dates provided by TimeTree v. 5 (Kumar et al. 2022) as the former will push the ringed seal crown age, as well as the divergence times of different ringed seal evolutionary lineages towards younger, rather than older, more controversial dates. The multi‐species time‐calibrated Bayesian analysis was characterised by high ESS values (Table S2) and pointed to a crown age for the sampled ringed seals at 538 kya (95% HPD interval = 322–741 kya) (Figure S2), which we used to rescale the root of the ringed seal mitogenome Bayesian phylogeny.

### 2.2 Skull Geometric Morphometrics

#### 2.2.1 Sampling

A total of 180 ringed seal skulls from the Baltic Sea (*N* = 49), Lake Ladoga (*N* = 28), Lake Saimaa (*N* = 24), East Greenland (*N* = 60) and the High Arctic (Northwest Greenland and Eastern Canadian Arctic) (*N* = 19) were measured, including 46 females, 62 males and 72 specimens of unknown sex (Figure 1; File S3). To avoid excessive allometric variation in the sample, only adult and subadult animals were included, with subadults being defined by centroid size close to the range of known adults, as described below. The skull specimens are held at the Natural History Museum of Denmark and the Museum of Natural History in Helsinki, Finland; none of them were sampled for the mitogenome analyses.

#### 2.2.2 Shape Analyses

Thirty‐one anatomical cranial landmarks were defined that could be unequivocally located and that were presumed to be homologous among all skulls (Figure S1, File S3). Three‐dimensional coordinates of the landmarks were registered with a Microscribe 3D digitizer. The raw landmark coordinates were run through the generalised least‐squares Procrustes superimposition (Rohlf and Slice 1990) using the MorphoJ program (Klingenberg 2011), which was also used for all initial analyses. The Procrustes procedure used here was amended to deal with the redundancy of data points caused by the symmetry of the vertebrate skull (Klingenberg et al. 2002). To exclude size‐related variation, all further analyses were performed on the residuals of a multivariate linear regression of skull shape (i.e., Procrustes coordinates) on size (i.e., the logarithm of centroid size, CS; the square root of the summed squared distances of all landmarks to the centroid of the configuration).

To ensure independence of vectors describing sexual and geographical differences, the directionality of a vector describing shape differences between males and females at each locality was compared to the vectors describing shape differences among areas. The respective vectors were defined by linear discriminant analysis vectors between males and females and linear discriminant analysis vectors between the five sampling areas. Finding that geographical differences were unrelated to sex (see below), it was decided to pool sexes for geographical comparisons to improve sample sizes and include specimens with unknown sex.

Multivariate comparisons were performed on the first 10 components of a principal components analysis (PCA) to reduce the number of variables relative to the number of observations in the smaller samples from Northwest Greenland and Canada, Lake Ladoga and Lake Saimaa. These 10 PCs accounted for 67% of the total variance in the dataset. All subsequent PCs each accounted for less than 3.5% of total variance. Shape differences between the five sampling areas were explored using the first 10 PCs with R v4.3 (R Development Core Team 2015). A canonical variates analysis with classification of each specimen to the most likely geographical area by jackknife cross‐validation based on Mahalanobis distance (Lachenbruch 1967) was used to assess the success rate of assignment of skulls to the area of origin by shape. Success rates were also assessed by jackknife reclassification of pairwise comparisons between Arctic, Baltic, Ladoga and Saimaa ringed seals, respectively. Hotelling's T^2^ tests were used for pairwise comparisons for statistically significant shape differences between all pairs of sampling areas. Student's *t* tests were used to compare centroid sizes between all areas. The variation of skull shapes within each area was also assessed by computing the Procrustes distance of each specimen to its group mean shape. Like the analysis of centroid sizes, Student's *t* tests were used to compare the levels of variation in skull shape between areas.

## 3 Results

### 3.1 Mitogenome Analyses

#### 3.1.1 Diversity and Differentiation

The mitochondrial haplotype (*H*_d_) and nucleotide diversity (π) of Baltic Sea ringed seals were surprisingly high, with almost every animal having a unique haplotype (Table 1). Lake Ladoga seals had relatively high haplotype diversity and low levels of nucleotide diversity, whereas Saimaa ringed seals were characterised by very low haplotype and nucleotide diversity, with only seven unique haplotypes found among a sample of 112 animals.

The estimates of pairwise genetic differentiation (*K*_ST_) were high and statistically significant for all comparisons between the Baltic Sea, Lake Ladoga, Lake Saimaa and Arctic ringed seals (Table 2). The highest level of pairwise genetic differentiation was found between Saimaa ringed seals and Baltic ringed seals, as well as between Saimaa and Ladoga ringed seals, whereas the lowest level was estimated between the Arctic and the Baltic Sea. Likewise, estimates of *d*_A_ were higher for Ladoga and Saimaa ringed seals and generally lower for comparisons including Baltic Sea ringed seals (Table 2). Within the Arctic, estimates of *K*_ST_ and *d*_A_ were close to zero, with only Ulukhaktok (Eastern Beaufort Sea, Canada) showing some level of genetic differentiation from other Arctic localities, although this was not statistically significant (Table S1).

**TABLE 2:** Mitogenomic pairwise genetic differentiation between Fennoscandian and Arctic ringed seals estimated by *K*_ST_ (above diagonal) and *d*_A_ (below diagonal).

	Atlantic Arctic	Baltic Sea	Lake Ladoga	Lake Saimaa
Atlantic Arctic		0.054	0.137	0.290
Baltic Sea	0.0013		0.151	0.513
Lake Ladoga	0.0040	0.0023		0.754
Lake Saimaa	0.0036	0.0066	0.0099	

*Note:* All *K*_ST_ estimates are statistically significant at *p* < 0.001.

#### 3.1.2 Haplotype Network and Phylogeny

**FIGURE 2:**
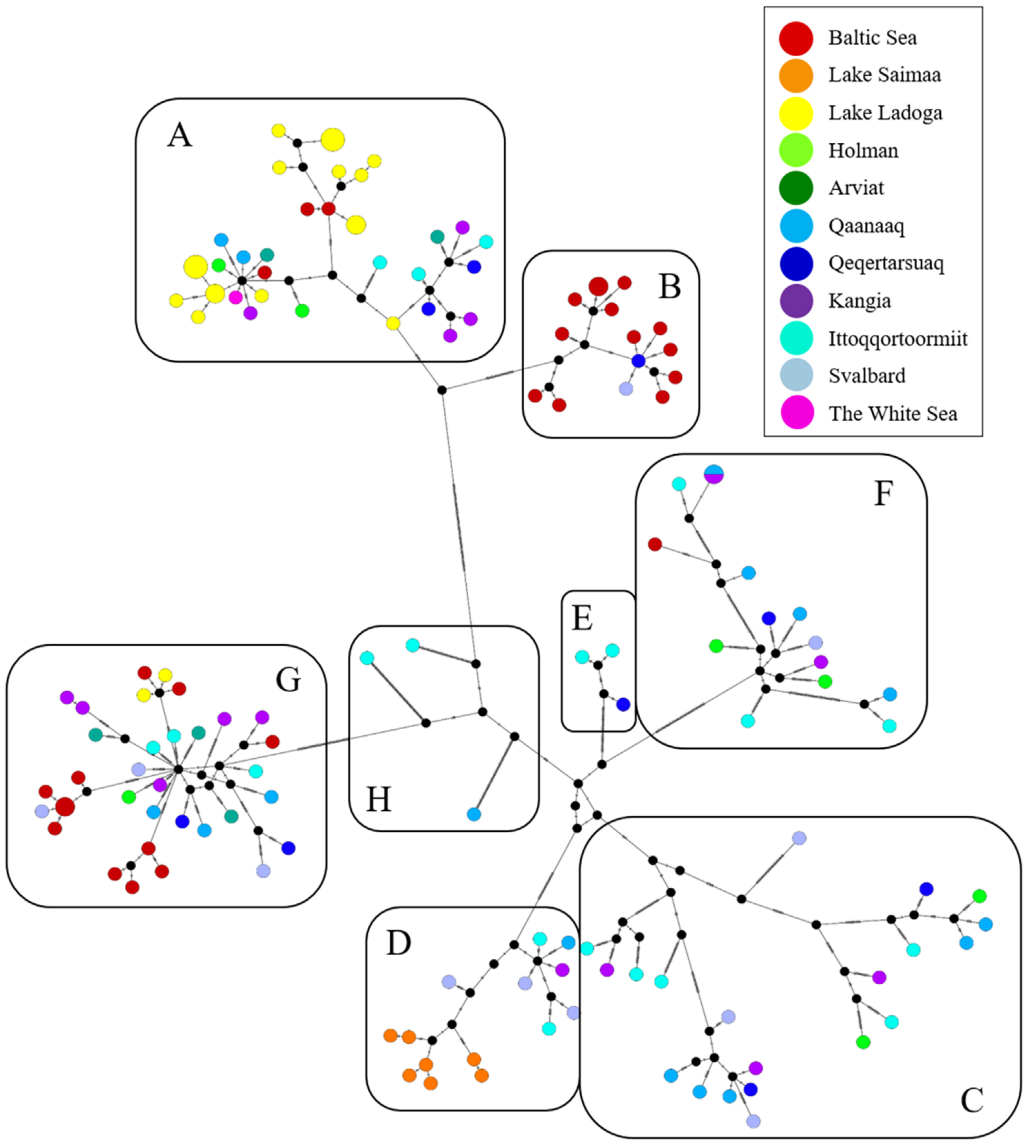
Ringed seal mitogenome median‐joining haplotype network illustrating the eight major lineages (A–H) found in the Atlantic Arctic, Baltic Sea, Lake Ladoga and Lake Saimaa. Colour and size of circles reflect sampling locality and relative abundance of each haplotype, respectively. Black bars on the branches denote segregating sites and inferred haplotypes not present in the data are represented by black circles. Note that for ease of illustration, we down‐sampled the data from Saimaa ringed seals from 112 animals to only include the seven unique haplotypes detected.

The median‐joining haplotype network suggested the existence of eight evolutionary lineages, consisting of six major and two minor lineages across the sample range (Figure 2). These include: Lineage A dominated by Ladoga haplotypes, but also containing Arctic and Baltic haplotypes; lineage B dominated by Baltic haplotypes and a few Arctic ones; lineages C and F with Arctic haplotypes; lineage D containing all the Saimaa haplotypes, as well as a few Arctic haplotypes at the base; a mixed lineage G with haplotypes from most sample regions, except Saimaa; and finally two minor lineages E and H consisting of Arctic haplotypes. Arctic ringed seals appear to occupy the central positions of the network, whereas Baltic, Ladoga and Saimaa seals occupy the tips, suggesting that Arctic haplotypes are ancestral.

**FIGURE 3:**
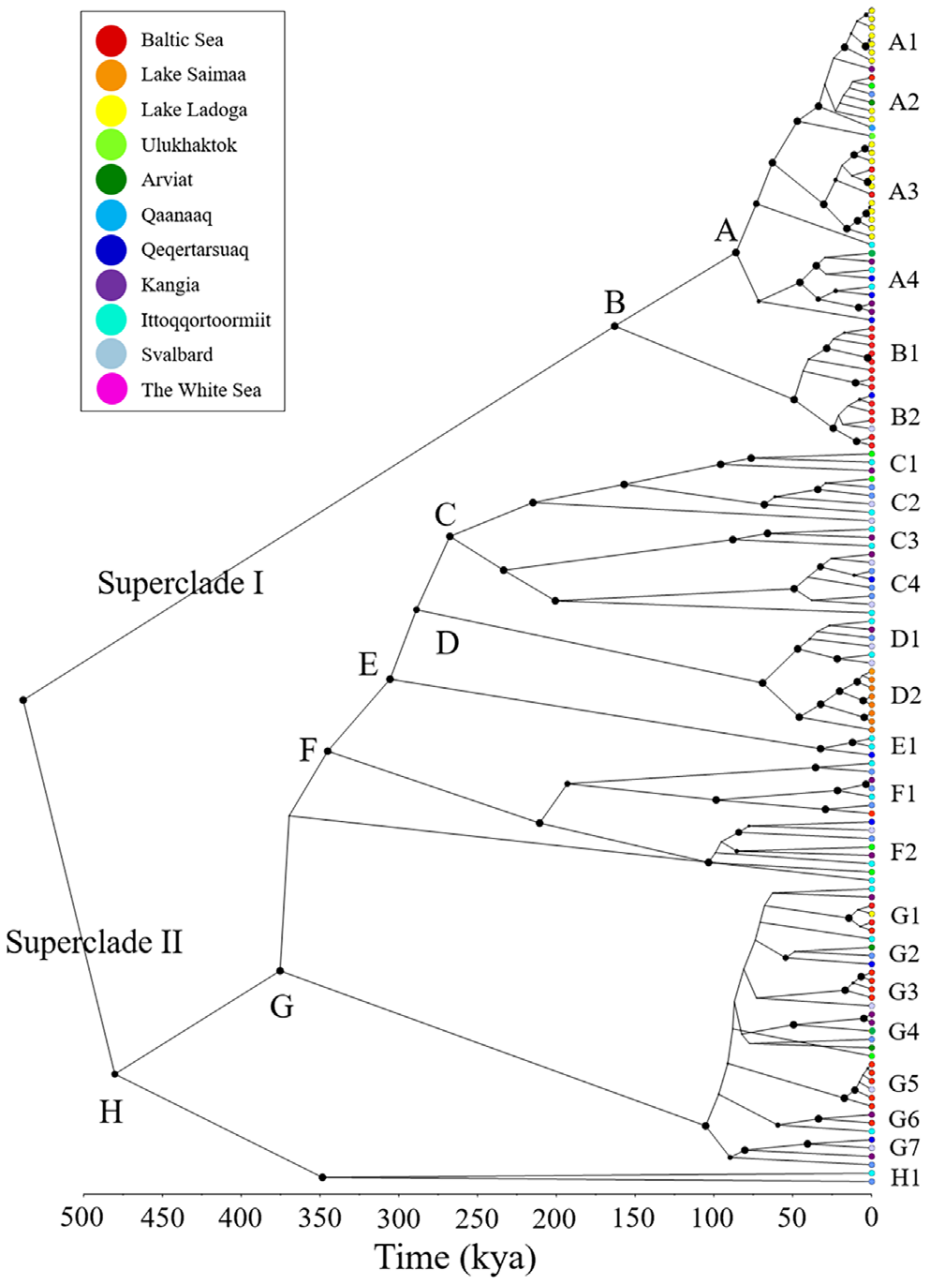
Bayesian phylogenetic tree based on 141 ringed seal mitogenomes. The root was rescaled to a crown age for ringed seals at 0.5385 Mya (95% HPD interval 0.3217–0.741 Mya) based on a multispecies time‐calibrated Bayesian phylogenetic analysis of eight phocid seal mitogenomes and 36 ringed seal mitogenomes representing major lineages (Figure S2). The analyses indicate the existence of eight evolutionary lineages (A–H). Most lineages have shallow internal branch lengths, except C and F. Sample IDs, branch support values and 95% HPD intervals on branch divergence time estimates are provided in Figure S3.

The ringed seal Bayesian and maximum likelihood phylogenetic analyses confirmed the existence of the ringed seal lineages identified in the haplotype network (Figure 3; Figures S3 and S4; Table S3). The crown age (root) estimated in the phocid multispecies phylogeny at 538 kya marks an early split between two major lineages I and II, within which the Arctic lineages C, E, F and H emerge 250–500 kya, and the remaining Arctic and Fennoscandian lineages A, B and D appear 150–50 kya. Mitogenome lineage G, comprising a mix of Arctic and Fennoscandian seals, diverged early from other lineages at circa 470 kya, but diverged into (sub)lineages much later at circa 100 kya. In Fennoscandia, the lineage D2 leading to Saimaa ringed seals diverged from Arctic ringed seals in lineage D1 about 70 kya and diverged into distinct haplotypes about 33 kya. The Ladoga seals (and a few Arctic and Baltic seals) in lineages A1‐A2 split from the Arctic lineage A3 about 86 kya. Notably, the A2 lineage, consisting exclusively of Ladoga and a few Baltic seals, but no Arctic ringed seals, emerged about 63 kya and diverged into distinct haplotypes about 30 kya. Baltic ringed seal haplotypes were assigned to multiple distinct lineages, but predominantly belong to lineage B, which diverged from other ringed seals about 163 kya. This group diverged further into lineages B1 and B2 about 49 kya. Lineage G was characterised by low internal resolution, except for three well‐supported lineages G1, G3 and G5 comprising mainly Baltic animals, which appear to diverge from Arctic seals in lineage G about 15–18 kya.

### 3.2 Skull Geometric Morphometrics

#### 3.2.1 Comparison of Shape Vectors

The vectors describing shape differences between males and females and the differences between the ringed seal distribution areas were largely independent. All vectors describing geographical differences among the five sample areas had angles between 85.7° and 98.6° (with 90° representing complete independence) when compared to the vector describing male–female differences, except for the pairs Saimaa–Ladoga (56.9°) and Saimaa–East Greenland (108.0°). A jackknife reclassification of skulls based on sex yielded a success rate of 63.9%, demonstrating very modest sexual dimorphism and large shape overlap between sexes. Thus, sexes were pooled for further analysis of shape.

#### 3.2.2 Geographical Differences in Skull Shape, Diversity and Size

Highly significant shape differences (*p* < 0.0001) were detected among all areas, except between Arctic localities (High Arctic vs. East Greenland), which had a more moderate *p*‐value (*p* = 0.013). These values were in turn reflected in the Procrustes and Mahalanobis distances among the regions, with the largest shape distances found between the Lake Ladoga and Lake Saimaa populations and the smallest distances between East Greenland and the High Arctic (Table 3).

**TABLE 3:** Ringed seal skull geometric morphometric distances among the five sampling regions, estimated by Procrustes (above diagonal) and Mahalanobis (below diagonal).

	High Arctic	East Greenland	Baltic Sea	Lake Ladoga	Lake Saimaa
High Arctic		0.017	0.020	0.032	0.031
East Greenland	1.940		0.017	0.025	0.031
Baltic Sea	5.040	3.570		0.028	0.030
Lake Ladoga	9.460	7.030	7.120		0.036
Lake Saimaa	9.800	8.590	8.120	11.300	

**FIGURE 4:**
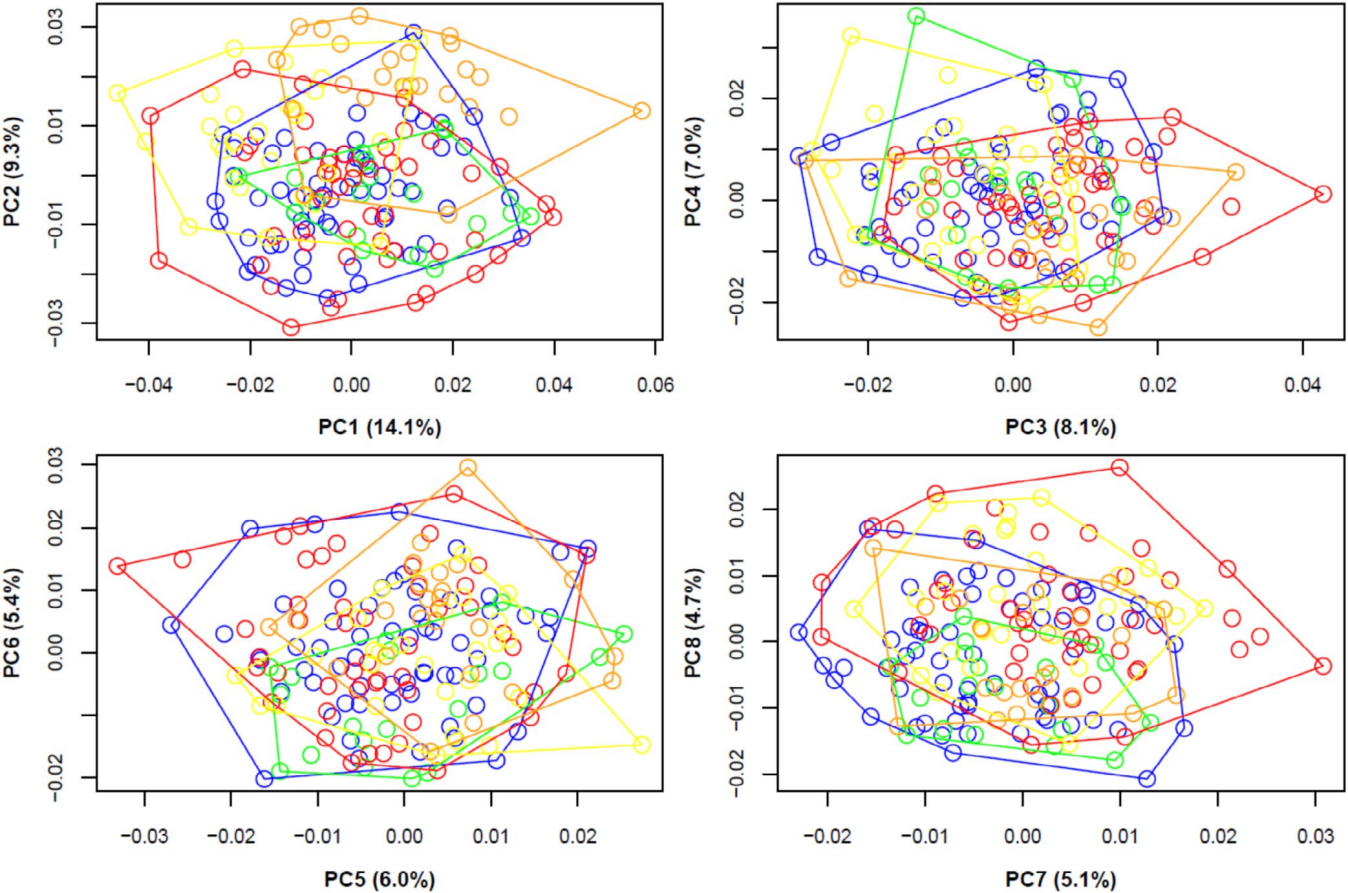
Morphospace representation of ringed seal cranial shapes using the first eight principal components, representing 58.7% of the residual variation after correcting for allometry. Symbol and convex hull polygon colour codes: Red = Baltic; orange = Saimaa; yellow = Ladoga; blue = East Greenland; and green = High Arctic.

The success of the reclassification of specimens to geographical areas of origin by shape using jack‐knife cross‐validation varied from 48% to 53% for East Greenland and the High Arctic, respectively, 59% for the Baltic Sea and 82% to 88% for Ladoga and Saimaa seals, respectively (Table 4). Assignment success was between 79% and 90% for all pairwise comparisons, with the lowest being between the Arctic and the Baltic ringed seal, where the more divergent seals in Saimaa and Ladoga were not involved (Table 5). These values reiterate that despite the highly statistically significant differences in skull morphology, there are substantial overlaps in shape between most of the sampled areas. This is also illustrated by plots of the first eight principal components, accounting for 58.7% of the total variance of the residual shape after correction for allometry (Figure 4). Subsequent PCs all accounted for < 4% of variance and did not show patterns related to geography. Differences between area‐specific shapes and the grand mean of shapes among areas were small (Figure S5). The morphological differences are most noticeable for the landlocked ringed seals, where Lake Ladoga skulls display a somewhat longer, laterally narrower, and dorsally compressed skull compared to the grand mean. Lake Saimaa skulls showed an opposite tendency, being somewhat shorter and dorsally expanded with slightly larger orbits compared to the grand mean.

**TABLE 4:** Number and proportion of ringed seal specimens correctly assigned to each geographical area by canonical variate analysis of skull geometric morphometric shape using Jackknife cross‐validation.

Origin	Assignment					
	High Arctic	East Greenland	Baltic Sea	Lake Ladoga	Lake Saimaa	Total
High Arctic	**10 (0.53)**	4 (0.21)	2 (0.11)	3 (0.16)	0 (0.00)	19
East Greenland	13 (0.22)	**29 (0.48)**	11 (0.18)	3 (0.05)	4 (0.07)	60
Baltic Sea	7 (0.14)	4 (0.08)	**29 (0.59)**	6 (0.12)	3 (0.06)	49
Lake Ladoga	1 (0.04)	1 (0.04)	0 (0.00)	**23 (0.82)**	3 (0.11)	28
Lake Saimaa	1 (0.04)	2 (0.08)	0 (0.00)	0 (0.00)	**21 (0.88)**	24
Total	32	40	42	35	31	180

*Note:* Bold values show specimens that were correctly assigned, regular font shows specimens that were incorrectly assigned.

**TABLE 5:** Proportion of ringed seal specimens that were correctly assigned to geographical area by linear discriminant analysis of skull geometric morphometric shape using jackknife cross‐validation in pairwise comparisons.

Comparison	Classification success
Arctic‐Baltic	78.9%
Arctic‐Ladoga	88.8%
Arctic‐Saimaa	90.3%
Baltic‐Ladoga	88.1%
Baltic‐Saimaa	86.3%
Ladoga‐Saimaa	84.6%

**FIGURE 5:**
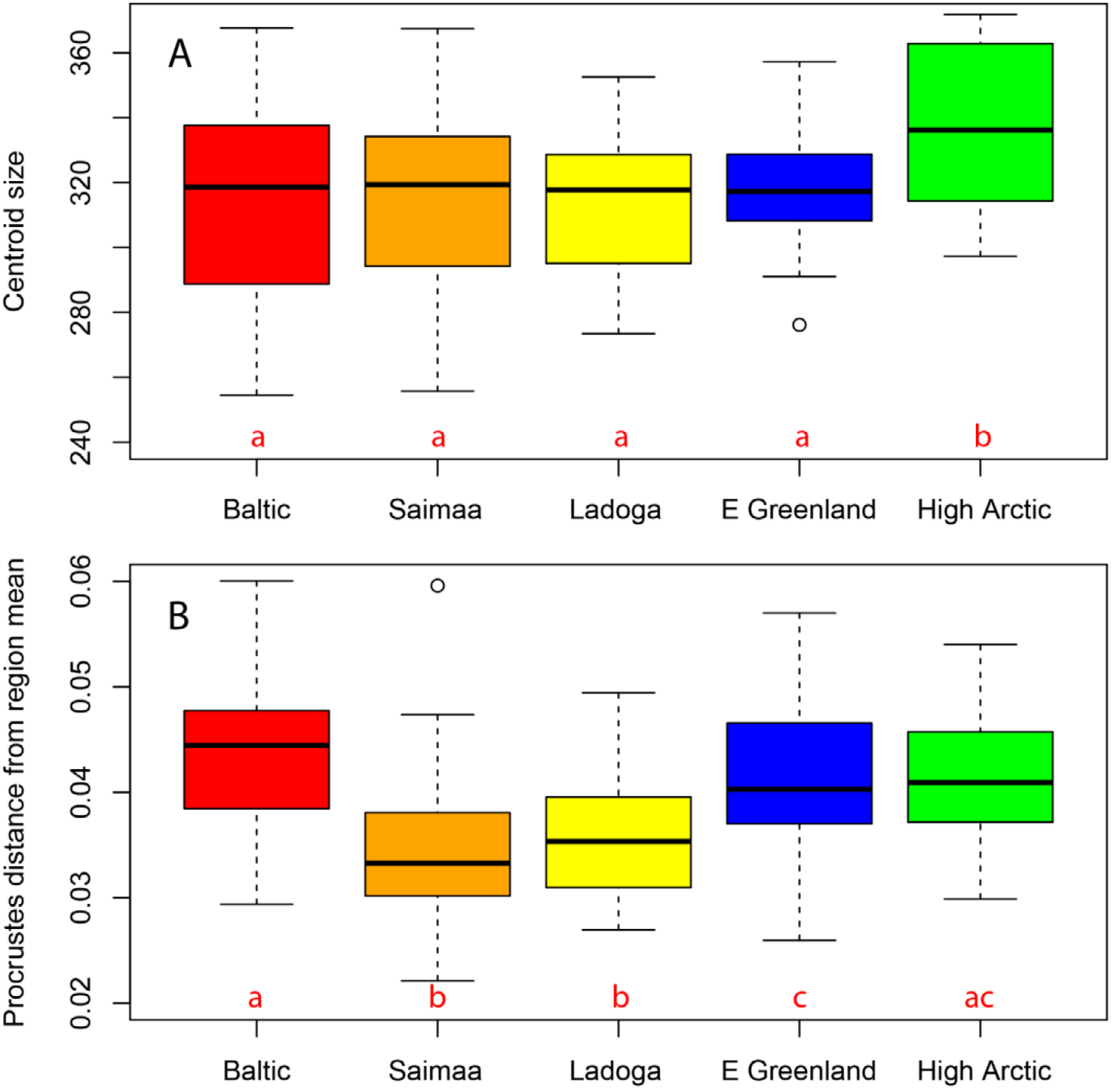
Size and shape variation in Fennoscandian and Arctic ringed seal skulls measured in terms of (A) centroid sizes and (B) Procrustes distances of specimens to the mean of the relevant sample. The bold horizontal line shows the median and the bottom and top of the box show the 25th and 75th percentiles, respectively. The vertical dashed lines (whiskers) show one of two things; either the maximum value or 1.5 times the interquartile range (roughly 2 standard deviations) of the data, whichever is the smaller. Points are outliers, defined as more than 1.5 times the interquartile range above the third quartile (below the first quartile). Same red letters signify regions that are not statistically significantly different from each other. Red = Baltic; orange = Saimaa; yellow = Ladoga; blue = East Greenland; and green = High Arctic.

The largest skulls were found in the High Arctic of Northwest Greenland and Northeast Canada, whereas the other areas did not differ much in average skull size (Figure 5A). The Baltic Sea sample showed the largest variation in skull size, whereas the landlocked Ladoga and Saimaa seals showed the lowest levels of variation (Figure 5B). The average shape variation within areas as defined by Procrustes distance to sample mean was larger than the between‐sample Procrustes distances, again highlighting modest geographic variation in ringed seal skull shapes.

## 4 Discussion

### 4.1 Fennoscandian Phylogeography Suggests Complex Origins

**FIGURE 6:**
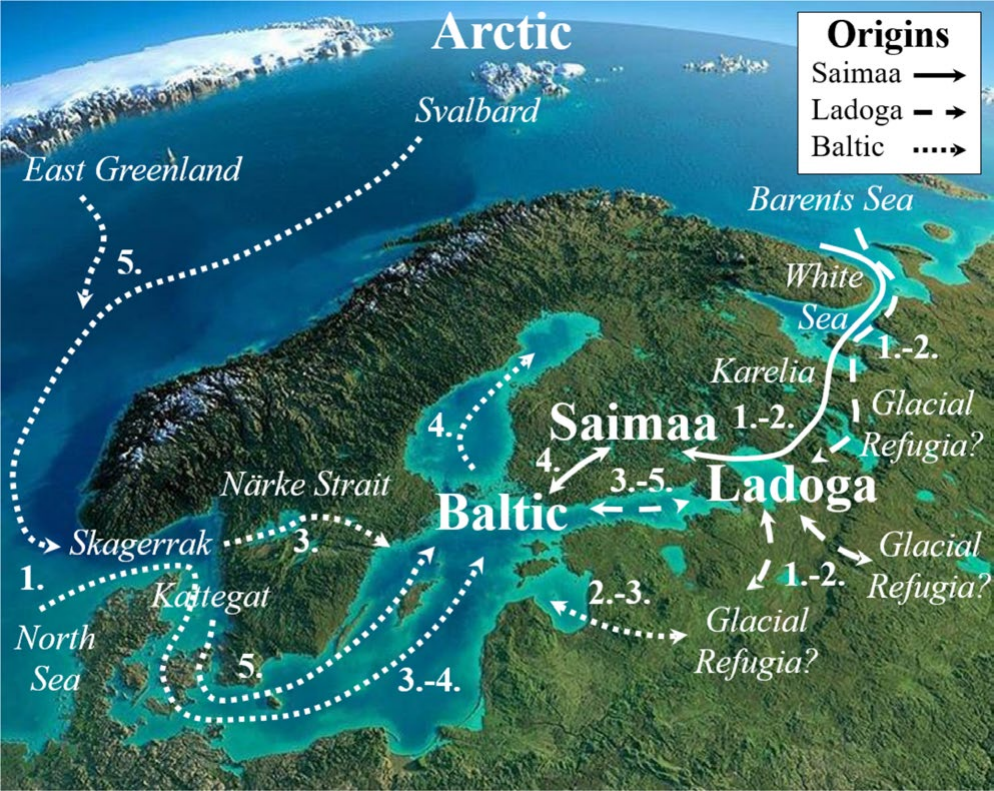
Conceptual map illustrating the hypothesised complex origins of Fennoscandian ringed seals through several colonisation events, routes and glacial refugia. Pre‐LGM (1.) and LGM (2.) events are hypothesised based on genetic and geological data presented here and/or in previous studies (see Discussion), whereas late Pleistocene (3.), early Holocene (4.) and present day (5.) events are well supported by zooarchaeological and genetic data.

The origin of Fennoscandian ringed seals has been the subject of much scientific investigation and debate (e.g., Davies 1958; Palo et al. 2001; Sommer and Benecke 2003; Valtonen et al. 2012; Martinez‐Bakker et al. 2013; Nyman et al. 2014; Ukkonen et al. 2014; Heino et al. 2023). In our mitogenome analyses, we detected multiple levels of phylogenetic structure with eight distinct evolutionary lineages, which largely grouped into two major lineages: I (lineages A–B) and II (lineages C–H). Fennoscandian ringed seals showed some phylogeographic patterning according to region, whereas no clear pattern was detected among Arctic ringed seals, with animals from Canada, Greenland and Svalbard scattered across all eight phylogenetic lineages. Our genetic data strongly point towards a more complex origin of Fennoscandian ringed seals than previously assumed, which we tentatively attribute to diversification predating arrival in Lake Ladoga, Lake Saimaa and the Baltic Sea, respectively, possibly through multiple colonisation routes and waves from glacial refugia at the edge of the Fennoscandian Ice Sheet (Figure 6).

#### 4.1.1 Saimaa Ringed Seals

Like previous studies, we found that the Saimaa seal was highly genetically (and morphologically) distinct from other Fennoscandian and Arctic ringed seals. It was characterised by high pairwise estimates of genetic differentiation and formed a single monophyletic lineage (D2) nested deep within multiple largely Arctic lineages (C, D1, E and F). Recent analyses by Heino et al. (2023) based on ~700 bp mtDNA control region data found multiple Baltic Sea haplotypes nested within Saimaa haplotypes, which may be attributed to periodic gene flow, e.g., during the Baltic Ice Lake stage; however, ours’ and most other studies find Saimaa to be monophyletic. Even if gene flow has occurred, most available data indicate that it has been at very low rates. Like us, Heino et al. (2023) found Saimaa ringed seals nested deep within Arctic haplotypes, which we interpret to mean that the Saimaa ringed seal evolutionary lineage originated during a single colonisation event from an Arctic‐like source (Figure 6). This contrasts with most other previous interpretations, which have pointed to a Skagerrak‐Kattegat origin. The exact source of these Saimaa seal ancestors remains obscure, potentially due to insufficient sample coverage from significant portions of the ringed seal's Russian range, the notable mitogenome homogeneity observed in Arctic ringed seals, or the possibility that the source population has become extinct over time. Regardless of its origin, evidence is mounting that the Saimaa seal's evolutionary lineage is substantially older than previously assumed. Recent nuclear genome analyses indicate that Saimaa ringed seals have followed a distinct demographic trajectory at least since the LGM and possibly up to 50–60 kya or older (Löytynoja et al. 2023). Similarly, demographic modelling using microsatellite markers suggested a Saimaa‐Baltic divergence c. 95 kya, although the authors interpreted this–at the time unexpectedly old divergence time estimate–as bias caused by the use of unusually high mutation rates (Valtonen et al. 2012). Moreover, a recent study of seal lice (*Echinophthirius horridus*) in Fennoscandian ringed seals supports a split of these from Arctic seal lice about 96 kya and indicates that seal lice in Saimaa seals have followed their own demographic trajectory for 60–80 ky (Sromek et al. 2024). Our mitogenome data suggest that lineages D1 and D2, consisting of Arctic and Saimaa haplotypes, respectively, diverged from other ringed seals about 289 kya, that D1 and D2 diverged about 69 kya, and that the single Svalbard haplotype at the root of D2 split at 46 kya, after which Saimaa seals comprise a monophyletic clade. Thus, we too find that the mitogenome lineage of the Saimaa ringed seal has a pre‐LGM origin. The length of the lineage (branch) leading to lineage D following its split from lineage C at 289 kya points to a long period of isolation and low abundance (bottleneck) before ultimately colonising Lake Saimaa.

We hypothesise that the Arctic ringed seals giving rise to the Saimaa evolutionary lineage (D2) could have colonised the region from the Barents Sea and White Sea through the Karelia seaway, which connected the Pre‐White Sea and the Pre‐Baltic Sea region, including Lake Ladoga and Lake Saimaa, during the Eemian interglacial (130–115 kya) (Funder et al. 2002; Lebas et al. 2021). Subsequently, these ancestors of the Saimaa seal could have maintained large viable populations at the north‐ and southeastern edge of the Fennoscandian Ice Sheet. This region was ice‐free during the Early Weichselian glaciation (109–71 kya) (Svendsen et al. 2004; Andren et al. 2011), and much of the eastern Baltic Sea, Karelia and White Sea region was characterised by a vast network of enclosed marine seas and ice‐dammed lakes up until the Middle Weichselian about 60–50 kya (Mangerud et al. 2004; Helmens and Engels 2010). Likewise, the period 55–35 kya was characterised by long climatic interstadials, in which time was sufficient to efficiently reduce the size of the Fennoscandian ice sheet (Kleman et al. 2021), and even the LGM 30–20 kya was characterised by large glacial lake systems at the ice margin (Hughes et al. 2016; Patton et al. 2017). At the end of the LGM (19 kya), the southeastern edge of the Fennoscandian Ice Sheet was characterised by a continuous presence of a vast system of ice lakes (Gorlach et al. 2017). Several of these ice lakes were similar in size to present‐day Lake Saimaa (4400 km^2^), and a few even similar to present‐day Lake Ladoga (17,700 km^2^) (Gorlach et al. 2017), and could thus easily have supported a viable ancestral Saimaa seal population that tracked the receding LGM ice sheet and its associated glacial lakes to ultimately colonise present‐day Lake Saimaa during the early Holocene. The unique genetic and morphological characteristics of Saimaa seals suggest little to no admixture with Baltic and Ladoga seals since the initial diversification of Saimaa ancestors from Arctic ringed seals. This observation is puzzling as the Baltic Sea, Lake Saimaa and Lake Ladoga periodically formed a continuous waterbody during the early Holocene, e.g. at the Baltic Ice Lake stage, but may owe to a strong breeding and/or moulting site fidelity, as documented in Saimaa, Baltic and to some extent Arctic ringed seals (Kelly et al. 2010; Koivuniemi et al. 2016; Yurkowski et al. 2016; Biard et al. 2022; Löytynoja et al. 2023).

#### 4.1.2 Ladoga Ringed Seals

Ladoga ringed seals were also characterised by high levels of genetic (and morphological) differentiation in our analyses, albeit not at the same levels as estimated for the Saimaa seal. Our results indicate that the origin of Ladoga ringed seals is more complex than previously assumed, comprising a mix of very different evolutionary lineages with possible distinct histories of refugia and colonisation (Figure 6). In lineage G1, two haplotypes are found in Ladoga seals, whereas the rest occur in Baltic and Arctic ringed seals. Consistent with earlier studies (Palo et al. 2001; Nyman et al. 2014), we interpret this as indicative of a post‐LGM (Holocene) colonisation and/or gene flow from the Baltic Sea, e.g. during the Baltic Ice Lake stage, during which present‐day Lake Ladoga and the Baltic Sea formed a combined waterbody (Vassiljev et al. 2011; Ukkonen et al. 2014). Such periodic gene flow with the Baltic may still be ongoing through the Neva River, yet is and has evidently not been of a magnitude to cause genetic and morphological homogenisation.

However, while the Ladoga seals in lineage G appear to be of post‐LGM origin, most other Ladoga seal haplotypes occur in two other lineages, A1 and A3, together with the mainly Arctic lineages A2 and A4 from which they diverged about 24 kya and at least 30 kya (but possibly up to 63 kya), respectively. Thus, similarly to the D2 haplotypes found in Saimaa seals, the A1 and A3 haplotypes in Ladoga seals appear to have an Arctic pre‐LGM origin, rather than a post‐LGM Baltic origin. The A lineage split from the B lineage about 163 kya and has a crown age of about 86 kya, and may thus have entered Fennoscandia from the Arctic via the White Sea and the Karelia Seaway during the Eemian interglacial and found refuge in enclosed seas and/or glacial lakes during the Weichselian and LGM, like hypothesised for the Saimaa seal. Alternatively, as discussed below, the A1 and A3 haplotypes in Ladoga seals may be traced to a cryptic northwestern European refugium.

#### 4.1.3 Baltic Ringed Seals

In contrast to Lake Saimaa and Lake Ladoga, where ringed seals are dominated by haplotypes from one and two lineages, respectively, the Baltic ringed seal carries haplotypes from four distinct lineages (A, B, F and G), suggesting that it has a mixed ancestry. The Baltic haplotypes in lineages A, F and G appear to be of relatively recent origin, diverging 20–10 kya from Arctic seals. This is in agreement with previous accounts on the post‐LGM colonisation of the Baltic Sea by ringed seals from the Skagerrak–Kattegat region (Figure 6). Indeed, while the earliest zooarchaeological remains of ringed seals in the Baltic Sea date to c. 10,440 kya, much older remains dating to at least 45,000 kya have been recovered in the neighbouring Skagerrak and Kattegat, documenting the existence of a ringed seal population in the region during the last glacial period (Ukkonen et al. 2014).

Not all Baltic ringed seals appear to share this origin. Most notably, nearly half of the Baltic ringed seals carry lineage B haplotypes, which are found almost exclusively in the Baltic Sea. Lineage B consists of two sublineages, B1 and B2, which diverged 49 kya, and itself appears to have separated about 163 kya from lineage A haplotypes found in Arctic, Ladoga, and a few other Baltic seals. The shape of the subtree leading to lineage B, characterised by a long branch length extending over > 100 ky from its split with lineage A to the divergence of the current B1 and B2 sublineages, indicates that the ancestral B haplotypes experienced a long period of isolation and low abundance (bottleneck) before colonising the Baltic Sea. Such isolation could have occurred in a cryptic refugium located at the edge of the Fennoscandian Ice Sheet (Figure 6). The North Sea (Sejrup et al. 2009; Patton et al. 2017; Panin et al. 2020), The Channel River (or ‘Fleuve Manche’) (Toucanne et al. 2009, 2023), or the extensive mosaic of glacial lakes described at the southwestern ice margin in present‐day Germany and the Netherlands (Lang et al. 2018) could all have supported such an isolated ringed seal population. Seals from either of these hypothesised glacial refugia could have gradually moved into the Pre‐Skagerrak–Kattegat to intermix with Baltic A, F and G haplotypes and colonise the Baltic Sea when it deglaciated during the Holocene.

### 4.2 Morphological Variation: Adaptation, Plasticity or Chance?

The results of our morphological analyses support the genetic data; the landlocked Saimaa and Ladoga ringed seals are the most morphologically divergent and showed the lowest levels of within‐group morphological variation. This complements the genetic results that the Saimaa seal has followed its own evolutionary trajectory and has likely been through bottlenecks. Saimaa seal skulls were shorter and dorsally expanded compared to the average skull shape across our overall sample, with somewhat larger eye orbits. As suggested in previous analyses (Amano et al. 2002), the distinct skull morphology of Saimaa seals–with relatively larger eyes and brains–may be adaptations for navigation in the murky and labyrinthine lake environment. In contrast, Ladoga seals display a somewhat longer, laterally narrower, and dorsally compressed skull compared to the grand mean. It is possible that these morphological adaptations arose prior to colonising Lake Saimaa and Lake Ladoga, respectively, perhaps during long periods of isolation in glacial refugia at the edge of the Fennoscandian Ice Sheet.

Baltic Sea ringed seals showed the largest within‐group variation in skull size and shape, corresponding with their relatively high mitogenome diversity; they were not highly distinct from Arctic seals. Multiple colonisation events of the Baltic region might have introduced diverse phenotypes and genotypes, boosting the Baltic ringed seal's overall genetic and morphological diversity. This diversity might have been maintained by Baltic ringed seals occupying multiple smaller pockets of suitable habitat with substantial variation in environmental conditions and prey bases in three main population centres in the Bothnian Bay, Finnish Archipelago Sea and Gulf of Riga, as well as a small, declining presence of ringed seals in the Gulf of Finland.

The skulls of High Arctic ringed seals had a wide size range but were, on average, larger than skulls from all other areas, in agreement with previous observations of body and skull size in the Arctic (Finley et al. 1983; Ferguson et al. 2018; Kovacs et al. 2021). This may reflect the large source area for this sample and perhaps also the latitudinal size cline found in eastern Canada (Ferguson et al. 2018, 2019). It is unclear whether this cline reflects phenotypic plasticity or natural selection. Recent genetic and ecological data suggest the existence of a distinct ‘Kangia’ ringed seal ecotype that inhabits the Ilulissat Icefjord in West Greenland (Rosing‐Asvid et al. 2023). The body size of these Kangia seals is as large as that of High Arctic ringed seals (Kovacs et al. 2021) and preliminary morphometric analyses indicate that their skulls are also distinctive (M.T. Olsen, unpublished data). It is likely that at least some of the morphological variation observed among Arctic ringed seals has a genetic background.

Interspecific variation in skull morphology of phocid seals (and many other vertebrates) is strongly linked to feeding ecology, with most species occupying distinct morphospaces and relatively easy to visually identify to species (Kienle and Berta 2016). However, the intraspecific variation in phocid skull morphology is often modest, and how this links to function is generally poorly understood (Galatius et al. 2022). Ringed seals are typically described as opportunistic foragers, using a combination of biting and suction to ingest a range of fish and invertebrate species (Siegstad et al. 1998; Holst et al. 2001; Kienle and Berta 2016; Scharff‐Olsen et al. 2018). This might buffer against divergence and adaptations in larger areas with a wide array of potential prey items, such as the Arctic and to some extent the Baltic Sea, while the landlocked seals in Lake Saimaa and Lake Ladoga may have adapted to a limited local prey diversity. Unfortunately, there is no investigation of the diet of Ladoga seals available to evaluate this (but see Geptner et al. 1988). However, Saimaa seals are known to have a relatively simple diet, with smelt ( *Osmerus eperlanus* ), ruff ( *Gymnocephalus cernuus* ) and perch (*Flerva fluviatilis*) making up more than 70% of prey (Auttila et al. 2015). We propose that this forced dietary specialisation might have been a driver of its morphological skull diversification. We note that marine populations of ringed seals, as well as other pinniped species, are known to occasionally visit and forage in rivers and adjoining lakes (Roffe and Mate 1984), so we would not expect an immediate strong selection on skull morphology caused by salinity changes when the Saimaa and Ladoga ancestors moved from marine to freshwater.

### 4.3 Status and Fate of Fennoscandian Ringed Seals?

Historical catch records indicate that the long‐term population size of Baltic Sea ringed seals was in the hundreds of thousands (Kvist 1991; Kokko et al. 1999), but that its population size was reduced to about 5000 animals in the 1960s–1980s due to hunting and organochloride pollution (Bergman and Olsson 1986; Harding and Härkönen 1999). Its abundance has increased in recent decades, currently numbering 20,000–30,000 animals, of which the majority reside in the Bothnian Bay, 1500 live in the Estonian part of the Gulf of Riga, 200–300 live in the Archipelago Sea, and less than 100 in the Gulf of Finland (HELCOM 2023). Nevertheless, Baltic ringed seals continue to face challenges and fall short of attaining good environmental status. New pollutants are emerging in the Baltic Sea ecosystem (Sonne et al. 2020b), pathogens might be introduced from sympatric seal and bird species (Sonne et al. 2020a), and overexploitation of fish stocks affects the quality and quantity of important prey species and, in turn, the nutritional status and reproductive success of Baltic ringed seals (Kauhala et al. 2019). Climate change is already severely affecting the southern Baltic distribution areas, where mild winters have greatly reduced the extent of sea ice with overlying snow for breeding lairs (Meier et al. 2022). The relatively high levels of phenotypic and genetic diversity reported here and elsewhere (Palo et al. 2001; Amano et al. 2002; Martinez‐Bakker et al. 2013) may provide some resilience to human impacts and environmental variation in Baltic ringed seals. Still, it is imperative that management and conservation measures are kept in place and adapted to address changes in the intensity of different impacts such as climate change. It has been debated whether Baltic ringed seals constitute a subspecies or a population (Palo et al. 2001, Amano et al. 2002, Martinez‐Bakker et al. 2013). For cetaceans, guidelines for taxonomy propose a classification of the population–subspecies boundary at *d*_A_ > 0.0006 for mitogenomes (Morin et al. 2023) and percent diagnosability (PD) > 80% for morphological data (Taylor et al. 2017). Using these criteria, our results indicate that Baltic ringed seals meet the subspecies criteria based on mitogenome data (*d*_A_ = 0.0013–0.0066), while diagnosability based on morphometrics was close to this threshold, at least in pairwise comparisons (PD = 78.9%–88.1%).

The landlocked Saimaa ringed seal is characterised by extremely low levels of diversity, with little variation in skull shape, and very low haplotype and nucleotide diversity in a sample of more than a hundred animals. These findings are similar to previous genetic studies (Palo et al. 2003; Valtonen et al. 2012; Nyman et al. 2014; Heino et al. 2023; Löytynoja et al. 2023; Sundell et al. 2023), which all suggest that Saimaa ringed seals have experienced bottlenecks during colonisation, and as a consequence of more recent human impacts and climate change (Kunnasranta et al. 2021). In Saimaa, dedicated conservation and management measures by researchers and volunteers have supported breeding efforts in years with insufficient snow and/or ice cover by shovelling snow piles and constructing floating man‐made ‘nest boxes’ with some success (Kunnasranta et al. 2021). In addition, rehabilitation, captive breeding and translocation (within Lake Saimaa and/or to other lake systems) are being considered to facilitate population growth and maintenance of genetic diversity (Sundell et al. 2023). However, due to its very low abundance estimated at only 400–500 animals, the most important conservation priority is to mitigate the risk of stochastic effects, including the spread of diseases and regulating fishing and recreation in key habitats and seasons (Kunnasranta et al. 2021). Saimaa ringed seals clearly meet the subspecies criteria proposed for cetaceans for mitogenome data (*d*_A_ = 0.0036–0.0099) and morphological distinctiveness (PD = 84.6%–90.3%), and we note that in some pairwise comparisons, it also meets the species criteria at *d*_A_ > 0.008 (Morin et al. 2023), while falling slightly below the species diagnosability criteria for skull morphometric data at > 95% (Taylor et al. 2017).

Ladoga seals are slightly more genetically and morphologically diverse than Saimaa seals, possibly due to a larger founding population, less severe impacts of human activities, and perhaps also occasional gene flow from Baltic ringed seals, as discussed above. Hunting of the Ladoga seal led to population declines from about 20,000 animals in the early 20th century to about 4000 animals in the 1970s, but since their protection in the 1980s, abundance increased to about 5000 animals in the 1990s and up to 8000 animals in 2012, with most occurring in the northern part of the lake (Sipilä and Hyvärinen 1998; Trukhanova et al. 2013). As with other Fennoscandian ringed seals, the Ladoga seal is threatened by bycatch, recreational activities and climate change through loss of ice and snow cover during the breeding season (Trukhanova et al. 2021). In our analyses, Ladoga ringed seals meet the subspecies criteria for both mitogenome (*d*_A_ = 0.0023–0.0099) and morphological data (PD = 84.6%–88.8%), and management should be undertaken accordingly.

Climate change is already affecting Fennoscandian ringed seals, particularly regarding declines in suitable breeding habitats with sufficient ice and snow cover, and these effects will only increase in the future (Meier et al. 2022). All Fennoscandian ringed seals, along with some Arctic counterparts (e.g., Northwest Greenland, Svalbard, White Sea and Bering Sea), resort to using land as a haul‐out platform during the summer when sea ice is unavailable (Lydersen et al. 2017, Olsen MT pers. obs., Gryba et al. 2021, Kunnasranta et al. 2021, Melnikov 2022, Svetochev et al. 2022, Trukhanova et al. 2024). Though breeding on land has only recently been reported for the Lake Ladoga ringed seal (Loseva et al. 2022), our and previous studies document the long‐term persistence of Fennoscandian ringed seals through interglacials and Holocene warm periods (e.g., Sommer and Benecke 2003; Ukkonen et al. 2014; Heino et al. 2023; Löytynoja et al. 2023). Thus, while land‐based breeding is likely associated with a substantial reduction in reproductive success, there is ground for some optimism for the future of Fennoscandian ringed seals, contingent upon the effective reduction of other anthropogenic pressures, including bycatch, disturbance of resting and breeding sites, competition for food, and pollution (Reusch et al. 2018; Sonne et al. 2020b).

## 5 Conclusions and Perspectives

Evidence is accumulating that the evolutionary histories of Fennoscandian ringed seals are more complex than previously assumed, characterised by several periods of isolation and connection during glacials and interglacials. Our finding that the diversification of Fennoscandian ringed seals predates the LGM is controversial given the lack of ringed seal zooarchaeological material from this period. However, prior to and during the LGM, we expect very few, if any, prehistoric human settlements that could account for the deposition of bone remains, and natural deposits are likely to be widely scattered and thus rarely unearthed. Our genetic analyses are limited in terms of their reliance on maternally inherited mitogenome markers and rather simplistic model assumptions for the ringed seal phylogenetic analysis. However, dating of the tree was conducted by comprehensive time‐calibrated multi‐species phylogenetic analysis, and the inferred divergence times from mitogenome data is supported by recent analyses of nuclear genomes (Löytynoja et al. 2023; Sromek et al. 2024) and historic mtDNA data (Heino et al. 2023). Importantly, we argue that the biology of the ringed seal makes it fully capable of surviving long glacial and interglacial periods in even small isolated glacial refugia. Evidently, ringed seals have been maintaining small isolated populations for millennia in the Saimaa and Ladoga lakes, as well as in Arctic glacial fjord systems (Rosing‐Asvid et al. 2023). The ringed seal's ‘sister species’–the Baikal seal ( *P. sibirica* )–might indeed have evolved from such an ancient relict ringed seal population (Sasaki et al. 2003; Palo and Väinölä 2006). The long‐term existence of lake populations is also known for harbour seals ( *Phoca vitulina* ), including the Lac des Loups Marins (Ungava) and Iliamna seals (Smith et al. 1994; Ferrer et al. 2024). Future research endeavours incorporating full nuclear genome data hold promise for providing additional insights into the evolutionary history of Fennoscandian ringed seals. Such studies may also include more detailed morphological and isotopic analyses to generate hypotheses regarding the role of foraging and other adaptations in the diversification process, contributing to a more comprehensive understanding of the complex evolutionary dynamics in ringed seals. Finally, we note that the Fennoscandian region holds other relict marine species (e.g., Funder et al. 2002), which may also be characterised by a more complex history of evolution and colonisation than previously assumed.

## Author Contributions

**Morten Tange Olsen:** conceptualization (lead), data curation (lead), formal analysis (lead), funding acquisition (lead), investigation (lead), methodology (lead), project administration (lead), resources (equal), supervision (lead), visualization (equal), writing – original draft (lead), writing – review and editing (lead). **Ari Löytynoja:** data curation (equal), resources (equal), writing – review and editing (equal). **Mia Valtonen:** data curation (equal), investigation (supporting), resources (equal), writing – review and editing (supporting). **Steen W. Knudsen:** formal analysis (equal), methodology (supporting), supervision (supporting), writing – review and editing (supporting). **Sofie Bang:** data curation (supporting), formal analysis (equal), investigation (supporting), writing – review and editing (supporting). **Casper Gunnersen:** data curation (equal), formal analysis (equal), funding acquisition (supporting), investigation (supporting), writing – review and editing (supporting). **Aqqalu Rosing‐Asvid:** data curation (supporting), funding acquisition (equal), resources (equal), supervision (supporting), writing – review and editing (supporting). **Steven H. Ferguson:** funding acquisition (supporting), resources (supporting), writing – review and editing (supporting). **Rune Dietz:** funding acquisition (equal), resources (supporting), writing – review and editing (supporting). **Kit M. Kovacs:** funding acquisition (supporting), resources (supporting), writing – review and editing (supporting). **Christian Lydersen:** funding acquisition (supporting), resources (supporting), writing – review and editing (supporting). **Jukka Jernvall:** data curation (supporting), funding acquisition (equal), resources (supporting), writing – review and editing (supporting). **Petri Auvinen:** data curation (supporting), funding acquisition (equal), investigation (supporting), resources (supporting), writing – review and editing (supporting). **Anders Galatius:** conceptualization (lead), data curation (equal), formal analysis (lead), funding acquisition (supporting), investigation (lead), resources (equal), supervision (lead), visualization (lead), writing – original draft (lead), writing – review and editing (equal).

## Conflicts of Interest

The authors declare no conflicts of interest.

## Supporting information


File S1.

File S2.

File S3.

Data S1.

## Data Availability

Mitogenomes and morphometric data are attached as supplementary files.
