# Supplementary material for: Complex Origins and History of the Relict Fennoscandian Ringed Seals
Source: Ecol Evol. 2025 Mar 4;15(3):e71067. doi: 10.1002/ece3.71067 (PMC11879273; doi:10.1002/ece3.71067)
Supplement: Supplementary file 4 — Data S1. [file ECE3-15-e71067-s004.docx]

**SUPPLEMENTARY MATERIAL**

**Supplementary Figure S1** Landmarks used for the geometric morphometric analyses


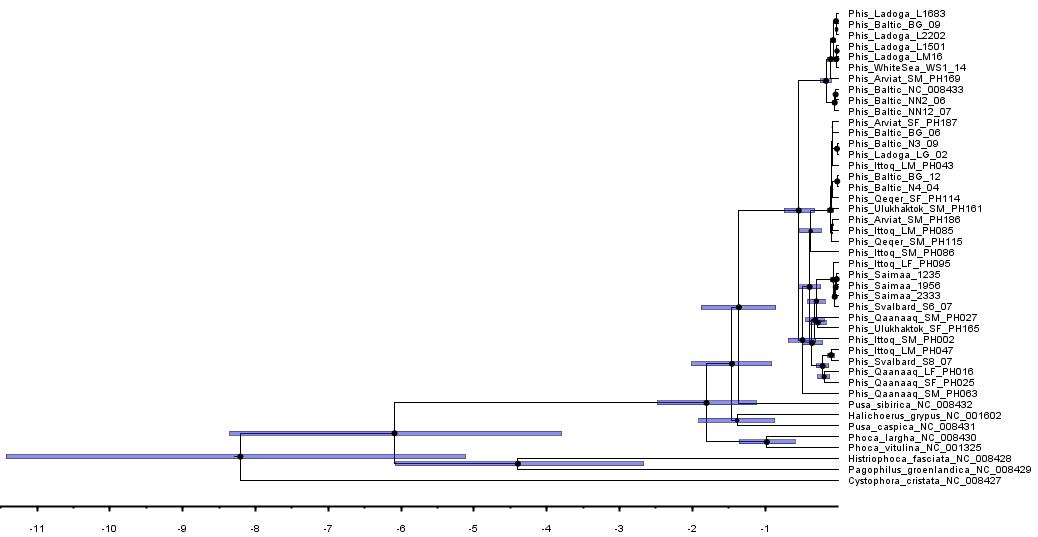


**Supplementary Figure S2** Multispecies time-calibrated Bayesian phylogenetic tree based on mitogenomes from 36 ringed seals representing major lineages in Figure 2 and Figure 3 with eight related phocid seal species included to time the tree and provide an estimate of ringed seal crown age at 0.5385 Mya (95% HPD interval 0.3217-0.741 Mya). The tree includes sample IDs, 95%HPD intervals on branch divergence time estimates, and node posterior support values above 0.90 illustrated by black circles. All nodes with posterior support <0.5 have been collapsed. A high resolution pdf is available as separate file.


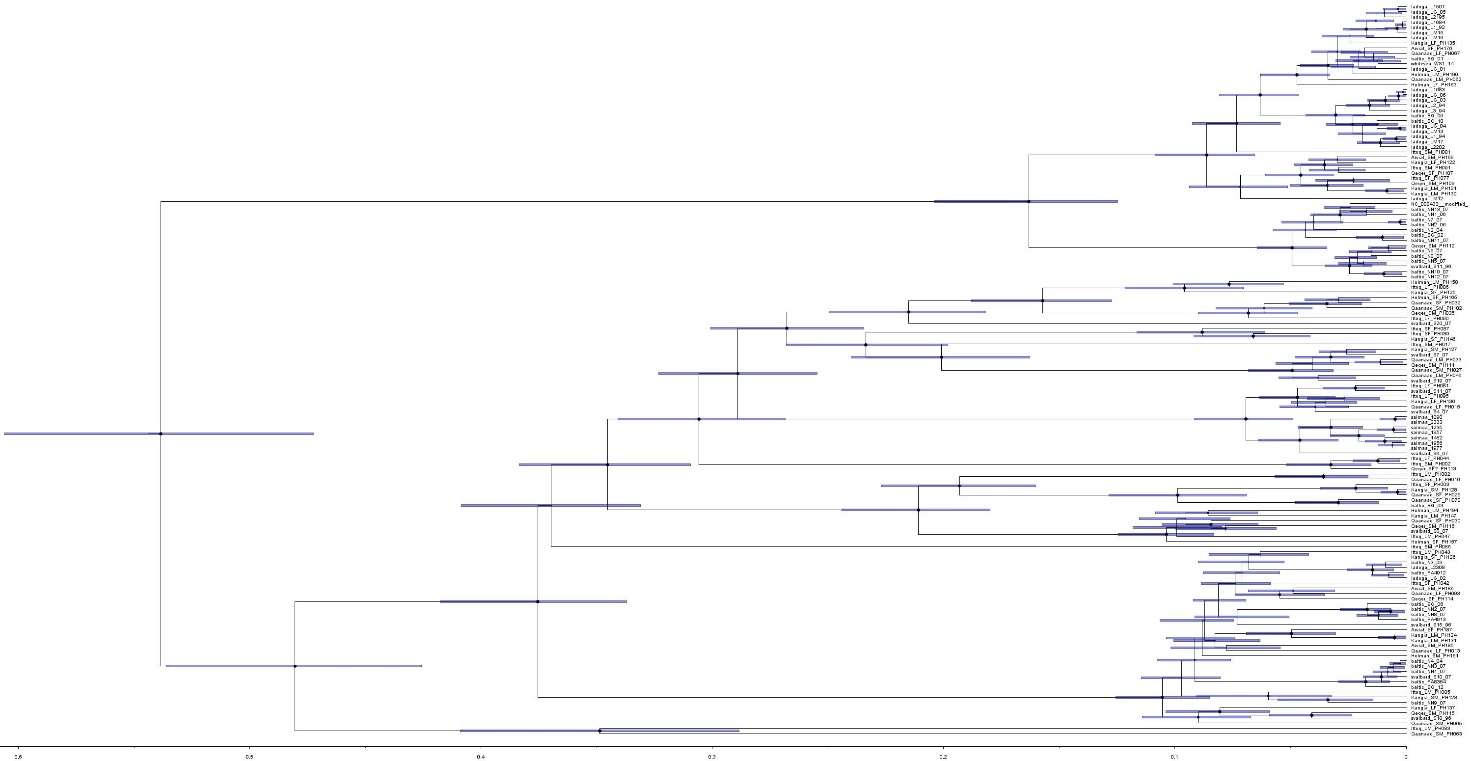


**Supplementary Figure S3** Time-calibrated Bayesian phylogenetic tree based on 141 ringed seal mitogenomes, representing populations the Atlantic Arctic, Lake Saimaa, Lake Ladoga and the Baltic Sea. The tree includes sample IDs, 95%HPD intervals on branch divergence time estimates, and node posterior support values above 0.90 illustrated by black circles. A high resolution pdf is available as separate file.


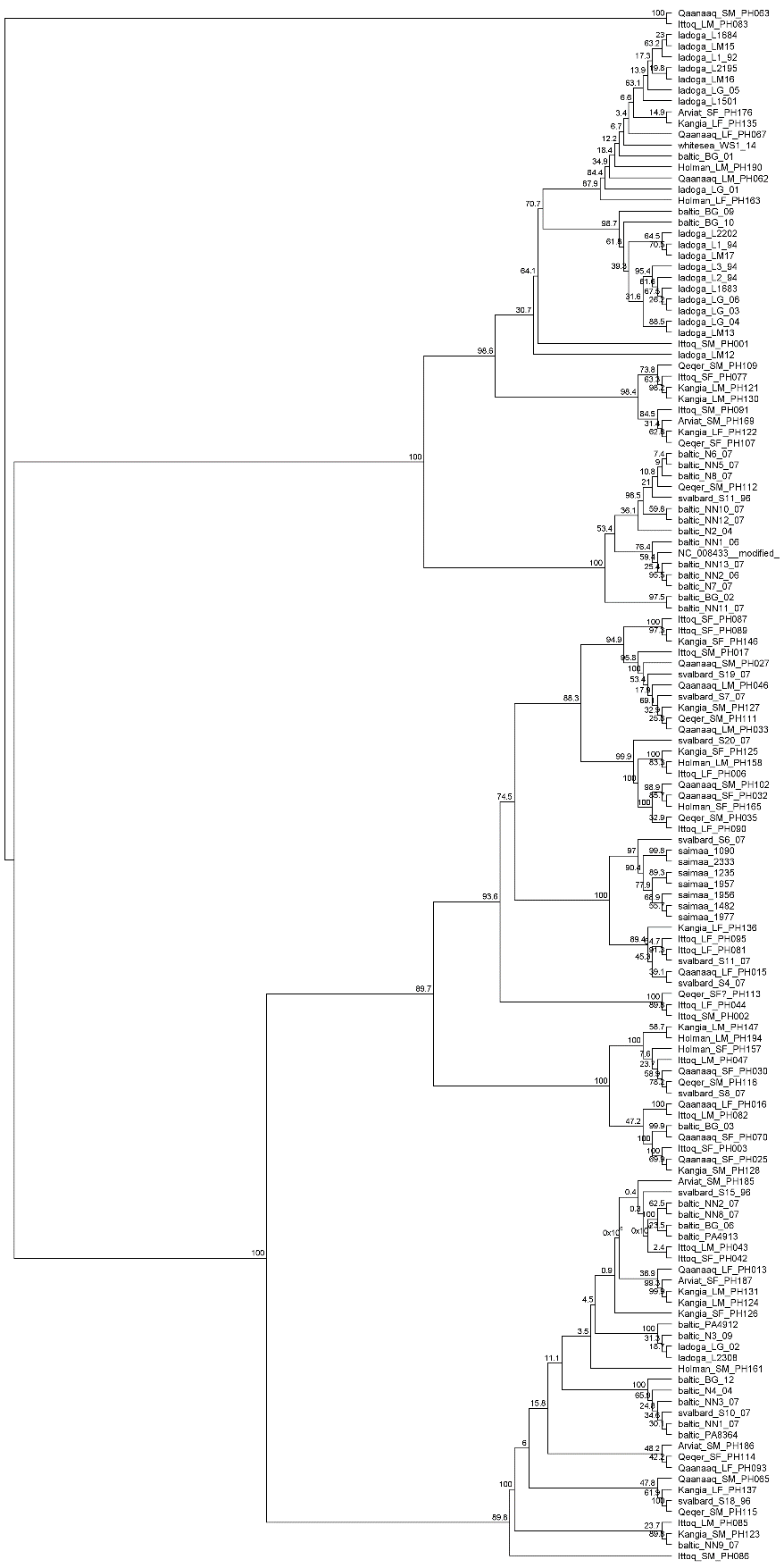


**Supplementary Figure S4** PhyML phylogenetic tree based on 141 ringed seal mitogenomes. The tree includes sample IDs and node support values. A high resolution pdf is available as separate file.


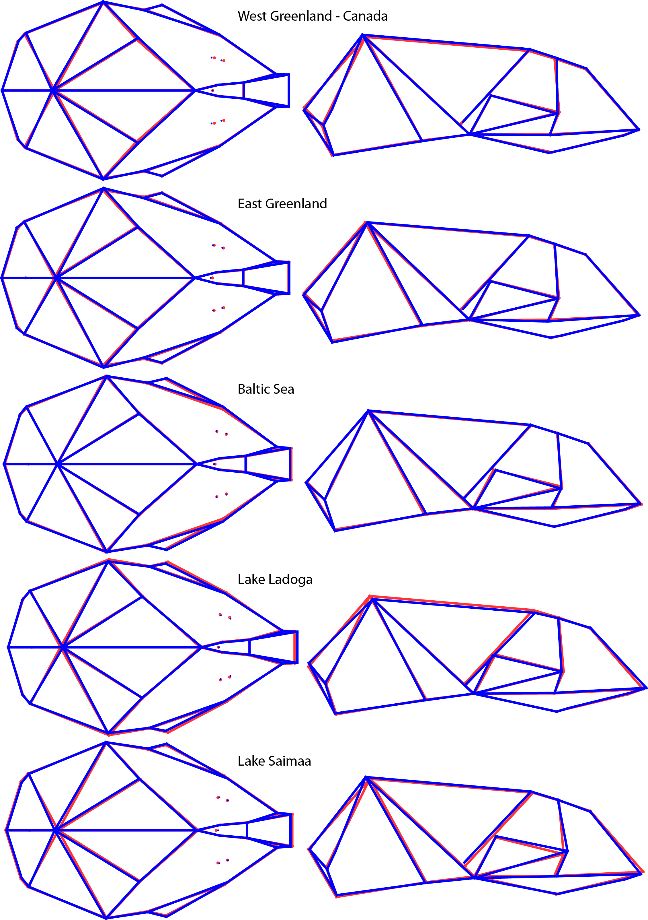


**Supplementary Figure S5** Mean area-specific shapes of ringed seal skulls from Baffin Bay, East Greenland, Baltic Sea, Lake Ladoga and Lake Saimaa (blue outline and markers) compared to the grand mean shape of all areas (red outline and markers). In general, Lake Ladoga ringed seals seem to have slightly longer snouts and more flattened skull, whereas Lake Saimaa ringed seals have shorter snouts, larger orbits and more compact skulls. Correction for allometric effects has been performed.

**Supplementary Table S1** Genetic differentiation between Arctic ringed seals estimated by *K*_ST_ (above diagonal) and *D*_A_ (below diagonal). None of the *K*_ST_ estimates were statistically significant at P<0.05.

|  | Ittoqqortoormiit | Qaanaaq | Qeqertarsuaq | Kangia | Ulukhaktok | Arviat | Svalbard |
| --- | --- | --- | --- | --- | --- | --- | --- |
| Ittoqqortoormiit |  | 0.0000 | 0.0000 | 0.0000 | 0.0000 | 0.0222 | 0.0000 |
| Qaanaaq | 0.0000 |  | 0.0000 | 0.0000 | 0.0000 | 0.0284 | 0.0000 |
| Qeqertarsuaq | 0.0000 | 0.0000 |  | 0.0000 | 0.0000 | 0.0000 | 0.0000 |
| Kangia | 0.0000 | 0.0000 | 0.0000 |  | 0.0000 | 0.0000 | 0.0000 |
| Ulukhaktok | 0.0000 | 0.0000 | 0.0000 | 0.0000 |  | 0.0103 | 0.0057 |
| Arviat | 0.0007 | 0.0007 | 0.0000 | 0.0000 | 0.0002 |  | 0.0348 |
| Svalbard | 0.0000 | 0.0000 | 0.0000 | 0.0000 | 0.0001 | 0.0007 |  |

**Supplementary Table S2** Summary statistics for the multi-species BEAST2 analysis

|  | mean | stderr of mean | stdev | variance | median | 95% HPD interval | ACT | ESS |
| --- | --- | --- | --- | --- | --- | --- | --- | --- |
| posterior | -31586 | 0.200 | 10.2 | 103.8 | -31586.9 | [-31605, -31565] | 3471 | 2593 |
| likelihood | -31541 | 0.193 | 6.0 | 36.4 | -31540.7 | [-31553, -31529] | 9261 | 972 |
| prior | -45 | 0.096 | 8.3 | 68.2 | -45.9 | [-60, -28] | 1217 | 7397 |

ACT = Auto-correlation time

ESS = Effective sample size

**Supplementary Table S3** Summary statistics for the ringed seal BEAST2 analysis

| Parameter | mean | stderr of mean | stdev | variance | median | 95% HPD Interval | ACT | ESS |
| --- | --- | --- | --- | --- | --- | --- | --- | --- |
| posterior | -23201 | 0.435 | 16.6 | 276.4 | -23201.0 | [-23233, -23168] | 24617 | 1463 |
| likelihood | -23806 | 0.338 | 14.2 | 200.8 | -23805.2 | [-23834, -23779] | 20436 | 1762 |
| prior | 604 | 0.293 | 11.7 | 137.1 | 604.5 | [580, 625] | 22498 | 1600 |

ACT = Auto-correlation time

ESS = Effective sample size

**Supplementary File S1** Nexus file with 246 ringed seal mitogenomes

**Supplementary File S1** Nexus file with 36 ringed seal and 8 related phocid seal mitogenomes

**Supplementary File S3** Txt file with ringed seal geometric morphometric data
